# Diagnostic CT-Based Palliative Radiotherapy Using kV Image Verification: A Prospective Feasibility Study

**DOI:** 10.3390/curroncol33100577

**Published:** 2026-09-25

**Authors:** Kelly Wei, Jason Hui, Bradford Gill, Sheri Grahame, Alexandra Guytingco, Wei Liu, Mitchell Liu, Eric Berthelet, Srinivas Raman, Elaine Wai, Shilo Lefresne

**Affiliations:** 1 Department of Radiation Oncology, BC Cancer, Vancouver, BC V5Z 4E6, Canada; kelly.wei@bccancer.bc.ca (K.W.);; 2 Faculty of Medicine, The University of British Columbia, Vancouver, BC V6T 1Z3, Canada; 3 Department of Radiation Therapy, BC Cancer, Vancouver, BC V5Z 4E6, Canada; 4 Department of Medical Physics, BC Cancer, Vancouver, BC V5Z 4E6, Canada

**Keywords:** diagnostic computed tomography, simulation-free radiotherapy, palliative radiotherapy, resource-limited settings, patient satisfaction

## Abstract

Patients receiving palliative radiotherapy (PRT) often experience substantial symptom burden and may face challenges attending multiple hospital visits. Planning treatment using an existing diagnostic computed tomography (dCT) scan instead of a dedicated computed tomography (CT) simulation may improve access to timely care. This prospective feasibility study evaluated a dCT-based planning workflow using kilovoltage (kV) image verification for patients with bone metastases, with potential applicability to resource-limited settings. Ten patients receiving conventional PRT (8 Gy in 1 fraction or 20 Gy in 5 fractions) with an eligible dCT acquired within 28 days were enrolled. As part of this feasibility study, all patients also underwent standard CT simulation, which served as the dosimetric comparator and fallback treatment pathway. Radiotherapy plans generated on dCT and CT simulation scans were compared for dosimetric quality, while treatment deliverability, patient satisfaction, and estimated carbon emissions were assessed. Seven of ten patients were successfully treated using the dCT workflow. The three remaining patients were transitioned to standard CT simulation treatments due to an uncorrectable dCT image rotation in the planning software (*n* = 1), scheduling constraints (*n* = 1), and a larger-than-anticipated difference in patient separation on the treatment unit (*n* = 1). Target coverage and organ-at-risk doses were comparable between dCT and CT simulation plans, satisfaction among dCT-treated patients was high, and potential avoidance of CT simulation was estimated to reduce carbon emissions by approximately 10 kg carbon dioxide equivalent (CO_2_e) per patient. These findings demonstrate that dCT-based planning with kV image verification is feasible, provides dosimetry comparable to conventional CT simulation, and warrants further evaluation as a strategy to improve access to timely PRT while reducing healthcare resource use and environmental impact.

## Simple Summary

Patients receiving palliative radiotherapy often have significant symptoms and may find multiple hospital visits burdensome. One way to reduce these visits is to plan treatment using an existing diagnostic computed tomography (CT) scan instead of requiring a separate planning CT scan. In this study, we evaluated a diagnostic CT-based planning workflow that uses standard X-ray imaging to confirm patient positioning before treatment. Most patients were successfully treated using this workflow, with radiotherapy plans comparable to those created using the conventional planning process. Treated patients also reported high satisfaction with the streamlined approach. By reducing the need for CT simulation, this workflow has the potential to shorten treatment pathways, reduce healthcare resource use and carbon emissions, and improve access to timely palliative radiotherapy, particularly in centres with limited resources.

## 1. Introduction

Patients referred for palliative radiotherapy (PRT) often have significant symptom burden, decreased performance status, and practical challenges related to travel and repeated hospital visits. Improving access to care while minimizing unnecessary appointments is therefore an important component of patient-centred PRT.

To improve access to care, diagnostic computed tomography (dCT)-enabled planning, also known as simulation-free radiotherapy, has been developed to eliminate the need for a separate computed tomography (CT) simulation [1,2,3,4]. Previous studies have demonstrated dosimetrically acceptable treatments while reducing referral-to-treatment time, hospital visits, and resource utilization compared with conventional planning pathways [3,4,5]. Pain response is equivalent, and patients report high satisfaction with this approach [4,6].

Recently, consensus recommendations have provided guidance regarding patient selection, workflow requirements, and multidisciplinary implementation of dCT-based PRT, recommending cone-beam CT (CBCT)-based image guidance for treatment delivery [7]. Although CBCT facilitates accurate treatment delivery, it may increase treatment-unit occupancy and require equipment or staffing that is not universally available. Furthermore, the incremental clinical benefit of CBCT in low-dose palliative treatments may be minimal. In addition to evaluating the impact of new initiatives on patient outcomes and workflows, it is also important to consider their environmental effects. This approach aligns with the triple bottom line framework, which acknowledges that public health is intrinsically dependent on planetary health. Therefore, assessing the environmental impact of clinical initiatives is a core metric for evaluating overall quality [8].

Whether selected patients can be safely treated using a pragmatic dCT-based workflow with surface landmark setup and kilovoltage (kV) image verification remains less well studied. We therefore developed a protocol using kV image verification for implementation in resource-limited settings. The primary aims were to evaluate deliverability and dosimetric acceptability of dCT-based plans. The secondary aims were to prospectively assess patient satisfaction and the anticipated carbon emissions and energy savings.

## 2. Materials and Methods

### 2.1. Trial Design

This single-arm prospective feasibility study was conducted at a single Canadian cancer centre and was approved by the joint University of British Columbia and BC Cancer Research Ethics Board (H23-03465). The trial was registered on ClinicalTrials.gov (NCT06235034). All patients provided written informed consent.

### 2.2. Participants

Patients offered conventional PRT for bone metastases located in the T5-lumbosacral spine, bony pelvis, or proximal femur were eligible. These sites were chosen due to their association with the most reproducible setup for dCT [1]. Patients were required to be able to comfortably maintain a supine treatment position. Patients requiring urgent radiotherapy, complex planning such as volumetric modulated arc therapy (VMAT), or treatment planning that needed to account for prior courses of radiotherapy were excluded. A dCT of the anatomic region to be treated was required within 28 days prior to enrollment. dCT suitability was assessed based on predefined imaging criteria, including minimal motion artefact and minimal high-density material, sufficient field of view, and an acquisition position similar to the proposed treatment position. dCT scans with slice thickness ≤ 2.5 mm were considered acceptable, consistent with the 2.5 mm slice thickness typically used for standard CT simulation.

### 2.3. Radiotherapy Planning and Treatment Delivery

All patients underwent CT simulation and tattoo placement as per standard-of-care. The CT simulation plan served as the comparator and was available for treatment if the dCT workflow was unsuccessful. The dCT was then registered to the CT simulation. The radiation oncologist (RO) contoured a region of interest (ROI) and placed either a single field or parallel opposed pair (POP) on the dCT, based on clinical exam findings and radiographic imaging. The spinal canal and/or bowel were contoured as organs at risk (OARs) when they were within the treatment field. Contours and fields were then copied over to the CT simulation and were reviewed by the RO. OAR revision was permitted to allow for accurate dosimetric comparison; however, if ROI revision was required, the patient would then proceed down the standard CT simulation pathway.

In an effort to reflect the most commonly used conventional PRT fractionations at our centre, prescriptions were either 8 Gy in a single fraction or 20 Gy in 5 fractions, at the treating RO’s discretion. Radiotherapy was planned on both the dCT and CT simulation scans using ARIA software (version 18.00.00) without inhomogeneity correction. Both radiotherapy plans were compared for dosimetric quality. The dCT plan was approved if at least 95% of the ROI was covered by 80% of the prescription dose (ROI V_80_ ≥ 95%) and if there was less than or equal to a 5% difference between the dCT and CT simulation for the following metrics: overall plan maximum dose (D_max_), mean and minimum dose (D_mean_, D_min_, respectively) to the ROI, and D_max_ of the spinal canal and bowel bag. The ROI V_80_ ≥ 95% threshold was selected as a pragmatic measure of adequate coverage for conventional, non-conformal PRT planned without inhomogeneity correction. Instances where these criteria were not met were considered protocol violations and reviewed on a case-by-case basis with the treating team to determine if proceeding was clinically safe. Additionally, if the dCT quality was insufficient, the patient proceeded with standard-of-care radiotherapy using the CT simulation plan.

Patients were gowned such that their CT simulation tattoos were not visible and were initially positioned using superior/inferior and left/right measurements from surface landmarks, typically the umbilicus or xiphisternum, to approximate the planned treatment isocentre. Treatment-day separation was estimated using the planned source-to-surface distance (SSD) and measured setup table height and compared with the planned separation from the dCT. Anterior and lateral kV images were acquired and matched to bony anatomy within the treatment field. Image-guided shifts were applied, with an additional verification kV image required for shifts >2 cm. The treating RO approved the final match before treatment. Separation differences >3 cm were reviewed by the treating RO on a case-by-case basis to determine clinical acceptability. If satisfactory positioning could not be achieved or more than three sets of kV images or repositioning attempts were required, the patient proceeded with the CT simulation plan. For patients receiving multiple fractions, temporary skin marks were added after the initial treatment to facilitate subsequent positioning. The study workflow is summarized in Figure 1.

### 2.4. Endpoints

The primary endpoints were the proportion of patients successfully treated using dCT-planned PRT and the dosimetric quality of the dCT plan compared with the CT simulation plan. Successful treatment was defined as delivery of the dCT plan following satisfactory treatment-unit setup and kV image verification according to the predefined criteria described above. Dosimetric quality was evaluated by comparing ROI coverage and predefined target and OAR dosimetric parameters between the dCT and CT simulation plans as highlighted in Figure 1. Reasons for failure were documented for patients who did not successfully complete the dCT workflow.

Secondary endpoints included patient satisfaction and anticipated carbon savings. Patient satisfaction was assessed using a six-question survey (four Likert-scale items and two optional free-text questions) administered immediately after treatment; time spent on the treatment couch was recorded as a surrogate measure.

Anticipated carbon savings were estimated for each patient completing the dCT workflow based on three components: (1) round-trip patient travel, calculated using postal-code-based distance estimates from Google Maps (web version) and a standard carbon dioxide equivalent (CO_2_e) conversion factor for mid-range petrol vehicles [9]; (2) CT simulation energy use (0.017 kg CO_2_e per scan, based on a British Columbia electricity grid emission factor of 14 g CO_2_e/kWh and a mean CT energy consumption of 1.2 kWh per scan [10,11]); and (3) CT simulation material use, estimated from a sample of five PRT patients, with material-specific CO_2_e values sourced from the literature [12,13,14,15,16,17,18,19,20,21].

### 2.5. Statistical Analysis

Patient demographics, tumor characteristics, treatment details, and dosimetric parameters were collected and analyzed using descriptive statistics. For paired dosimetric comparisons between dCT and CT simulation plans, differences were calculated on a per-plan basis (CT simulation—dCT) and summarized as the median of paired differences with range. Patient satisfaction was assessed with a 5-point Likert scale and summarized descriptively. All analyses were conducted using Excel (version 16.97).

## 3. Results

### 3.1. Patient Characteristics

Ten patients were accrued from May 2024 to April 2025. Patient demographics are summarized in Table 1. Nine patients had a single treatment site, while one patient had three sites, resulting in a total of 12 treatment sites.

### 3.2. Workflow Delivery

Of the ten patients enrolled, seven successfully completed the dCT-based planning and treatment workflow, representing nine treatment sites: five spinal sites involving the thoracolumbar (*n* = 2), lumbar (*n* = 2), and lumbosacral (*n* = 1) spine; three bony pelvic sites involving the sacrum, acetabula/pelvic rami, and sacroiliac joint; and one proximal femoral site. Among patients treated using the dCT workflow, the median treatment couch time was 9.8 min (range, 6.3–15.8), and a median of 1 kV image set (range, 1–2) was acquired per treatment site. The median difference between planned dCT separation and treatment-day separation measured on the treatment unit was 2 cm (range, 0.3–3.8 cm) (Table 1). The remaining three patients received radiotherapy using their standard CT simulation plan (Table 2). One patient’s dCT was unsuitable for planning due to an uncorrectable rotation in the diagnostic image when imported into the planning software. Another patient was withdrawn after planning and received standard-of-care treatment because of logistical constraints in the treating oncologist’s schedule. The remaining patient did not proceed with the dCT plan because the measured separation on the treatment unit differed from the dCT by 4 cm, which was estimated to increase the delivered dose by approximately 7%. No patient failed the dCT pathway because acceptable bony alignment could not be achieved on kV imaging.

### 3.3. Dosimetric Parameters

Median ROI coverage (V_80_) was 100% for both dCT and CT simulation plans. ROI, overall plan, and OAR dose metrics were similar between dCT and CT simulation plans (Table 3). One protocol violation involved exceeding the comparison criteria for bowel D_max_ (>5% difference between the dCT and CT simulation plans), which occurred with an 8 Gy single-fraction prescription. In this case, the bowel D_max_ was 121% (9.7 Gy) on dCT and 112.2% (9 Gy) on CT simulation, due to differences in patient separation. The absolute bowel D_max_ remained well below the institutional constraint used for more conformal treatment planning. Given the small absolute difference (0.7 Gy) and palliative intent, the discrepancy was considered clinically acceptable following RO review, and the dCT plan was approved. A second protocol violation involved ROI D_mean_, which was 107.3% (8.6 Gy) on dCT versus 97.9% (7.8 Gy) on CT simulation for an 8 Gy single-fraction prescription. Given the high target coverage and small absolute dose difference (0.8 Gy), the discrepancy was considered clinically acceptable following RO review, and the dCT plan was approved.

### 3.4. Patient Satisfaction

All seven participants who completed the dCT workflow completed the satisfaction survey (Figure 2). Overall, patients reported high satisfaction with the dCT workflow, with all participants indicating they would recommend the approach to other patients. One participant disagreed that the treatment experience was comfortable; however, in the free-text response, this participant attributed their discomfort to pre-existing hip and groin pain.

### 3.5. Estimated Carbon Emissions

Patients who completed the dCT workflow travelled a combined round-trip distance of 169 km (median 12 km per patient; range, 4–85 km), associated with 57 kg CO_2_e (median 4 kg CO_2_e per patient; range, 1–29 kg CO_2_e) [9]. The CT simulation process contributed 12 kg CO_2_e in total (1.7 kg CO_2_e per scan), primarily from materials (11.9 kg) including masks, gloves, gowns, sheets, needles, sanitizer, and antiseptic wipes [12,13,14,15,16,17,18,19,20,21]. CT simulation energy usage accounted for 0.12 kg CO_2_e in total (0.017 kg per scan). Total estimated emissions across the cohort were 69 kg CO_2_e (mean 10 kg CO_2_e per patient; range, 3–31 kg CO_2_e).

## 4. Discussion

This prospective feasibility study demonstrates that dCT-based planning for selected patients with bone metastases is deliverable using kV image verification, while achieving dosimetry comparable to standard CT simulation. Among patients who successfully completed the dCT workflow, satisfaction was high. The dCT pathway also has the potential to decrease carbon emissions and waste associated with PRT. These findings support further evaluation of dCT-based planning with kV image verification as a potential strategy to expand access to PRT, particularly in resource-limited settings.

Three patients in the study did not receive dCT treatment and were treated using the standard-of-care CT simulation plan, illustrating distinct challenges related to dCT suitability and anatomical/setup differences (Table 2). One was the first patient enrolled. The requirement for double planning on both the CT simulation and dCT, along with on-unit verification by the RO, was more time-consuming than standard-of-care practice, particularly for the first trial patient. The patient was treated with the standard CT simulation plan to avoid treatment delays due to logistical constraints in the treating RO’s schedule, rather than concerns with the dCT plan. The second patient’s diagnostic image had uncorrectable rotation and could not be adjusted in the ARIA planning software. This issue is highlighted by Roderick et al., who propose that it may be correctable using MIMVista software [22]. The third patient’s measured separation on the treatment unit was 4 cm less than planned on the dCT, estimated to result in a 7% higher delivered dose. The treating RO did not believe this was clinically appropriate and opted to treat with the CT simulation plan.

Separation differences remain an important consideration for simplified dCT workflows. In our standard practice, differences between the separation on CT simulation and separation measured on the treatment unit greater than 1.5 cm trigger an RO review to determine whether replanning is required. Five of nine treatment sites in our pilot exceeded this threshold, likely reflecting body contour differences between the curved dCT couch and flat treatment couch. Other groups have addressed this effect by using a Vac-Lok that mimics the dCT couch curvature [1,4,22], while others recommend adding tolerance ring structures with CBCT to assess contour changes and tissue depth [22]. Although separation differences can influence delivered dose, the clinical significance depends on the absolute D_max_, anatomic location of the hot spot, anticipated need for retreatment, and patient prognosis. Careful patient selection and physician review of separation discrepancies may therefore allow kV-based workflows to remain clinically feasible for selected palliative patients.

The recently published international Delphi consensus recommendations for dCT-based radiotherapy implementation recommend three-dimensional (3D) image guidance for initial treatment verification, reflecting both anatomical variation and the limited evidence supporting simpler image verification approaches [7]. Our study evaluated a kV image-guided workflow with the aim of developing a low-resource approach to delivering low-dose, non-conformal PRT that was clinically sufficient. Although CBCT is available at many centres, this technology was originally designed to allow the delivery of highly conformal radiotherapy with precision. When designing clinical workflows for simple palliative treatments, each component of the treatment pathway should be evaluated to ensure it provides meaningful clinical benefit while minimizing unnecessary resource utilization and burden to patients.

One such cost of CBCT is time spent on the treatment couch, which carries both operational and environmental implications. In our study, kV image verification was associated with a median treatment couch time of approximately 10 min. Lee et al. reported 45 min linear accelerator (LINAC) occupancy for a CBCT-guided simulation-free workflow [5]. As such, pathways requiring prolonged CBCT-guided treatment slots may have significant implications for treatment-unit operations and may burden palliative patients with significant bony pain. In the same study, prolonged LINAC occupancy increased overall life-cycle carbon emissions despite a substantial reduction in operational emissions achieved by eliminating patient travel associated with CT simulation [5]. Operational gains and the patient benefits of avoiding CT simulation could therefore be eroded by downstream image verification requirements. These findings suggest that kV-based workflows may preserve the clinical and environmental benefits of dCT-based radiotherapy while minimizing additional treatment-unit utilization.

Our findings also complement the Delphi consensus recommendations for dCT-based radiotherapy implementation [7]. Similar to the recommendations, our study focused on patients receiving conventional PRT to the thoracic/lumbar spine and pelvis, supporting the feasibility of this approach. The Delphi panel also recognized that, when appropriately trained, radiation therapists may contribute to patient identification and workflow quality assurance [7]. Building on these recommendations, radiation therapists may have an expanded role in identifying potentially eligible patients and streamlining referral into dedicated palliative radiation oncology programs (PROPs).

PROP clinics have historically offered same-day consultation, CT simulation, and treatment as a strategy to improve access [23,24,25]. However, such workflows still require time between CT simulation and treatment for contouring, planning, and quality assurance. O’Neil et al. demonstrated that patients who received dCT-enabled planning spent significantly less time in the cancer centre than those who received same-day CT simulation and treatment planning (0.4 h vs. 4.7 h) [4]. Reducing time spent at the centre is particularly valuable for unwell patients, who are also the patients most likely to require premedication, nursing support, and other departmental resources. Integrating dCT-based planning into existing PROP clinics may therefore enhance their patient-centred and operational benefits rather than replace them.

This feasibility study has several limitations. It was conducted at a single centre with a small cohort. It was also performed alongside the standard CT simulation pathway, with all patients undergoing CT simulation and tattoo placement. Therefore, the study evaluated the technical feasibility and deliverability of dCT-based planning rather than a fully simulation-free clinical pathway and could not assess the effects of a dCT-based treatment protocol on treatment access or resource utilization. By design, our protocol relied on kV image verification rather than routine CBCT, limiting generalizability to more complex treatments or those that incorporate CBCT-based planning. Patient satisfaction was assessed immediately following treatment only among the seven patients who successfully completed the dCT workflow; therefore, these findings may overestimate overall acceptability. As the dCT pathway is implemented clinically, the proportion of patients who do not successfully receive dCT-based treatment should be regularly monitored as a quality metric to identify challenges and inform ongoing improvement. For patients unable to receive dCT-based treatment, efforts should be made to obtain an urgent CT simulation for planning to avoid unintended treatment delays. Following clinical implementation, metrics such as referral-to-treatment time, patient-reported outcomes, resource utilization, and carbon emissions should continue to be assessed. Further studies could explore implementation in dedicated PROP clinics and radiation therapist-led identification of eligible patients.

In conclusion, this pilot study demonstrates the feasibility of delivering selected PRT using a dCT-based workflow with kV image verification. Although the cohort was small, the workflow achieved clinically acceptable dosimetry, suitable patient setup and treatment delivery, and appeared acceptable to patients. This approach may be particularly valuable in resource-limited settings, centres without routine CBCT access, or clinical situations where reducing LINAC treatment time is a priority. For appropriately selected patients, dCT-based PRT with kV image guidance has the potential to improve access to timely PRT while reducing resource use and environmental impacts.

## Figures and Tables

**Figure 1:**
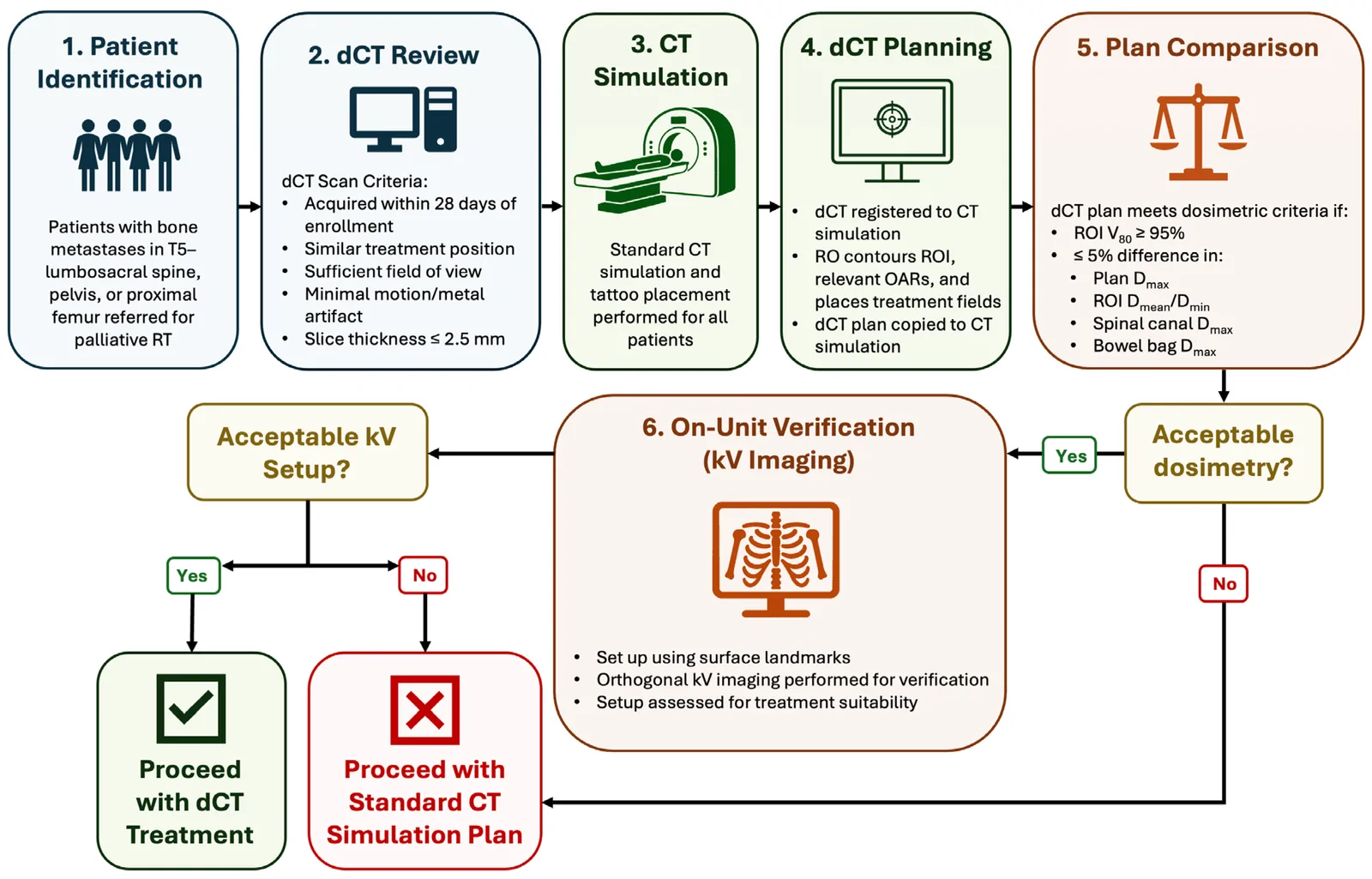
Diagnostic CT-based palliative radiotherapy workflow. Abbreviations: CT, computed tomography; dCT, diagnostic computed tomography; D_max_, maximum dose; D_mean_, mean dose; D_min_, minimum dose; kV, kilovoltage; OAR, organ at risk; RO, radiation oncologist; ROI, region of interest; RT, radiotherapy; V_80_, percentage of the ROI receiving at least 80% of the prescription dose.

**Figure 2:**
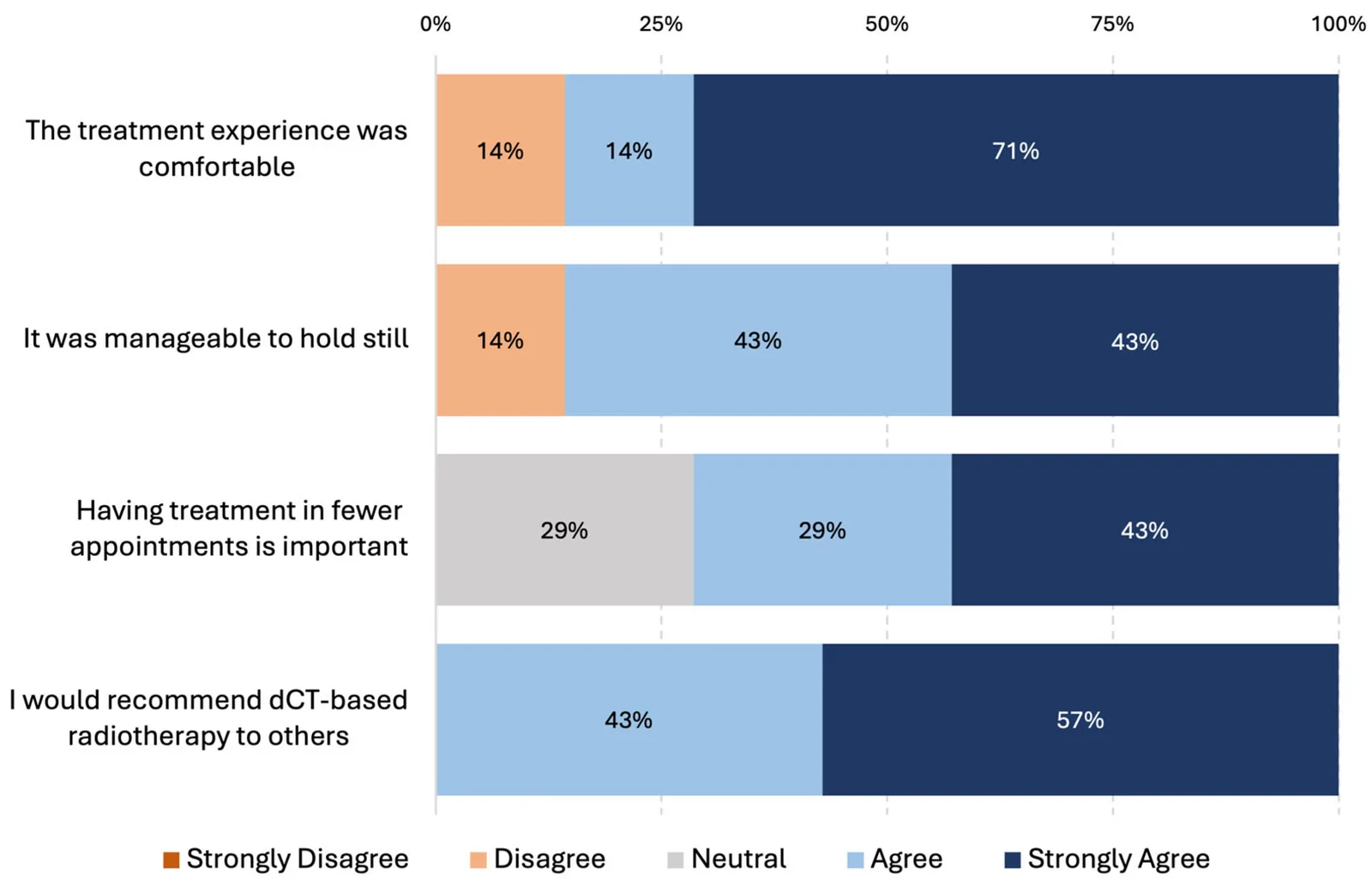
Patient satisfaction with the dCT-based radiotherapy workflow (*n* = 7).

**Table 1:** Patient, treatment, and workflow characteristics.

Patient, Treatment, and Workflow Characteristics		
	Characteristics	*n* (%) or Value
Median Age, Years (range)		74 (56–91)
Sex	Female	3 (30%)
	Male	7 (70%)
Primary Cancer	Prostate	5 (50%)
	Breast	2 (20%)
	Lung	1 (10%)
	Renal	1 (10%)
	Unknown primary	1 (10%)
Treatment Location *	Spine	6 (50%)
	Bony pelvis (non-femur)	4 (33%)
	Proximal femur	2 (17%)
Dose and Fractionation	8 Gy in 1	9 (90%)
	20 Gy in 5	1 (10%)
Patients Completing the dCT Workflow		7 (70%)
Median number of kV image sets **		1 (1–2)
Treatment Couch Time (min) **		9.8 (6.3–15.8)
Separation Difference (cm) **		2.0 (0.3–3.8)

* Patient characteristics are reported for ten patients with twelve treatment sites. ** Reported among seven patients successfully treated using the dCT workflow with nine treatment sites. Abbreviations: dCT, diagnostic computed tomography; kV, kilovoltage.

**Table 2:** Unsuccessful dCT workflow cases and potential mitigation strategies (*n* = 3). Abbreviations: CT, computed tomography; dCT, diagnostic computed tomography; D_max_, maximum dose; RO, radiation oncologist.

Unsuccessful dCT Workflow Cases			
**Workflow Challenge**	Category	Reason	Potential Mitigation
Image rotation	Image suitability	dCT rotation could not be corrected in planning software	Use image-registration software capable of correcting image rotation.
RO scheduling	Workflow logistics	Trial-specific dual planning and on-unit RO verification exceeded scheduled time; CT simulation plan used to avoid treatment delay	Omit dual planning and on-unit RO verification outside the clinical trial setting.
Separation discrepancy	Anatomical/setup discrepancy	Treatment-unit separation differed from dCT by 4 cm, estimated to increase delivered dose by 7%	Use Vac-Lok to reproduce dCT couch curvature. Consider clinical significance of dosimetric difference: D_max_, hot spot location, and patient prognosis.

**Table 3:** Dosimetric comparison between dCT and CT simulation plans for successfully treated sites (*n* = 9) among seven patients. Abbreviations: CT, computed tomography; dCT, diagnostic computed tomography; D_max_, maximum dose; D_mean_, mean dose; D_min_, minimum dose; ROI, region of interest; V_80_, percentage of the ROI receiving at least 80% of the prescription dose.

Dosimetric Comparison (*n* = 9 Sites)			
	dCT (Median, Range)	CT Simulation (Median, Range)	Difference * (Median, Range)
ROI V_80_ (%)	100% (100–100%)	100% (100–100%)	0% (0% to 0%)
ROI D_min_	97.7% (92.5–102.6%)	97.9% (92.2–101.7%)	0.5% (−2.3% to 2.6%)
	7.8 Gy (7.4 Gy–20.5 Gy)	7.8 Gy (7.4 Gy–20.3 Gy)	0 Gy (−0.2 Gy to 0.2 Gy)
ROI D_mean_	104.5% (99.8–108.9%)	102.4% (97.9–109.1%)	0% (−9.4% to 3%)
	8.4 Gy (8.0 Gy–21.2 Gy)	8.2 Gy (7.8 Gy–21.8 Gy)	0 Gy (−0.8 Gy to 0.6 Gy)
Plan D_max_	111.6% (105.7–120.4%)	109.4% (105.2–116.8%)	−1.5% (−4.9% to 0.2%)
	8.9 Gy (8.5 Gy–24.1 Gy)	8.8 Gy (8.4 Gy–23.4 Gy)	−0.1 Gy (−0.7 Gy to 0 Gy)
Spinal Canal D_max_	106.9% (104–112.7%)	105.4% (102.7–112.2%)	−1.3% (−2% to −0.2%)
	8.6 Gy (8.3 Gy–9.0 Gy)	8.4 Gy (8.2 Gy–9.0 Gy)	−0.1 Gy (−0.2 Gy to 0 Gy)
Bowel D_max_	108% (43.1–121%)	106.4% (41.3–113.4%)	−3% (−8.8% to 0.5%)
	8.6 Gy (3.4 Gy–22.6 Gy)	8.5 Gy (3.3 Gy–21.7 Gy)	−0.2 Gy (−0.9 Gy to 0 Gy)

* Median difference represents paired per-plan differences (CT simulation—dCT).

## Data Availability

The data presented in this study are available from the corresponding author upon reasonable request. The data are not publicly available due to privacy and ethical restrictions.
